# Long-term mortality rate and clinical outcomes associated with femoro-popliteal drug–coated balloon angioplasty and drug-eluting stents in chronic limb-threatening ischaemia: an analysis of the BASIL-3 RCT

**DOI:** 10.1093/bjs/znaf251

**Published:** 2025-11-24

**Authors:** Matthew Popplewell, Jack Hall, Lewis Meecham, Gareth Bate, Lisa Kelly, Jon J Deeks, Catherine A Moakes, Andrew Bradbury, the BASIL-3 Investigators, A Ganeshan, H Zayed, R Davies, A Saratzis, I Chetter, S Butterfield, S Goode, J Metcalfe, P Mezes, S Hobbs, N Goyal, J Patel, M Caruna, T Rashid, N Das, N Arya, P Chong, S Habib, R White, G Antoniou, A Davies, A Rogers, S Dindyal, N Arestis, G Weir, P Davey, R Das, G Stansby, R Eifell, J Asquith, L Wijesinghe, A Chaudhuri, J Sathianathan, N Thayur, F Ahmad

**Affiliations:** Black Country Vascular Network, Dudley Group of Hospitals, Dudley, UK; Department of Applied Health Sciences, University of Birmingham, Birmingham, UK; Birmingham Clinical Trials Unit, Department of Applied Health Sciences, University of Birmingham, Birmingham, UK; Department of Vascular Surgery, University Hospital of Wales, Cardiff, UK; Department of Vascular Surgery,University Hospitals Birmingham NHS Foundation Trust, Birmingham, UK; Department of Vascular Surgery,University Hospitals Birmingham NHS Foundation Trust, Birmingham, UK; Department of Applied Health Sciences, University of Birmingham, Birmingham, UK; Birmingham Clinical Trials Unit, Department of Applied Health Sciences, University of Birmingham, Birmingham, UK; Department of Vascular Surgery, University of Birmingham, Birmingham, UK

## Abstract

**Introduction:**

In recent years there have been a plethora of new endovascular devices have entered the market, including paclitaxel (PTX) drug-coated balloons (DCB) and drug-eluting stents (DES) for treating patients with chronic limb-threatening ischaemia (CLTI). There have been concerns that the use of PTX is associated with increased all-cause mortality rate in this patient population.

**Methods:**

In the BASIL-3 trial (ISRCTN14469736) UK patients with CLTI were randomized (1:1:1) to receive femoro-popliteal (FP) plain balloon angioplasty (PBA; with or without bailout bare metal stenting (BMS), DCB angioplasty (DCBA) (with or without BMS), or primary DES. Here, data from the DCBA and DES arms have been pooled into a single ‘drug technologies’ (DT) group and compared with PBA ± BMS. The primary outcome was overall survival (OS). Secondary outcomes included amputation-free survival (AFS), major amputations, major adverse limb events, major adverse cardiovascular events, reinterventions, and 30-day mortality and morbidity rates.

**Results:**

Four hundred and eighty-one participants were randomized (PBA: *n* = 160; DT: *n* = 321). At a median follow-up in survivors of 5.6 years, OS was similar between the pooled DT and PBA groups (adjusted hazard ratio (HR): 0.83; 95% c.i.: 0.64 to 1.07). There was no evidence of a statistically significant difference in AFS between the groups (adjusted HR: 0.84; 95% c.i.: 0.66 to 1.06), or other secondary outcomes.

**Conclusions:**

This further pooled analysis of the BASIL-3 RCT does not support the notion that the use of drug-eluting technologies, when compared to plain balloon angioplasty, increases all-cause mortality rate, or has other clinically important adverse effects, when used in patients with CLTI.

## Teaser

In the BASIL-3 RCT, when chronic limb-threatening ischaemia participants receiving drug technologies (drug-coated balloons and drug-eluting stents) were pooled and compared to those who received plain balloon angioplasty there was no evidence of a significant increase in mortality. Similarly, there were no other adverse secondary outcomes in those randomised to drug technologies.

## Introduction

Chronic limb-threatening ischaemia (CLTI) is the most severe form of atherosclerotic peripheral arterial disease (PAD) and is characterized by ischaemic pain and/or tissue loss (ulceration and/or gangrene)^1^. CLTI patients have a poor quality of life, and the great majority require revascularization to prevent amputation and prolong life. Endovascular interventions have become increasingly popular for the management of CLTI as they offer a less invasive alternative to surgical revascularisation, which may be associated with lower peri-procedural mortality and morbidity rates, albeit at the expense of reduced durability and so increased reintervention^2,3^ .

In the UK, plain balloon angioplasty (PBA), with or without bailout bare metal stenting (BMS), has long been considered the standard of care in those CLTI patients who are offered endovascular intervention in preference to vein bypass. In more recent years the use of drug-coated balloons (DCB) and drug-eluting stents (DES), which release antiproliferative drugs such as paclitaxel (PTX) directly into the arterial wall with the aim of reducing restenosis and maintaining the target artery pathway (TAP), have become used more frequently. Despite these potential benefits, the evidence supporting the clinical and cost effectiveness of these PTX devices when compared to PBA in patients with CLTI has been limited^4^.

To address this gap in the evidence base, following a research recommendation by the UK National Institute of Health and Care Excellence (https://www.nice.org.uk/guidance/cg147), the UK National Institute of Health Research Heath Technology Assessment (NIHR HTA) programme funded the BAlloon *versus* Stenting in severe Ischaemia of the Leg-3 (BASIL-3) RCT (ISRCTN14469736) to directly examine the clinical and cost effectiveness of femoro-popliteal (FP) DCB angioplasty (DCBA) (with or without BMS) and DES to PBA (with or without BMS) in patients with CLTI requiring a FP, with or without an infrapopliteal (IP), endovascular revascularization^5^ . In BASIL-3 no significant clinical benefit was observed for either DCBA (with or without BMS) or DES when compared to PBA (with or without BMS) in terms of amputation-free survival (AFS), mortality rate, or limb salvage^6^. Most of the participants allocated to DCBA or DES in BASIL-3 received PTX devices. One patient received a sirolimus-coated DCB and eight received everolimus DES.

The BASIL-3 trial initially opened to recruitment in January 2016 but was paused in December 2018 following the publication of a meta-analysis that showed that the use of PTX was associated with increased mortality rate at 3 and 5 years when compared to PBA^7^. The majority (4133/4663, 89%) of patients in this meta-analysis had intermittent claudication (IC) rather than CLTI, and individual patient-level data were not available. The UK Medicines and Healthcare Regulatory Authority (MHRA) convened an expert advisory group (EAG) (whose members were entirely independent of the BASIL-3 investigators) to review the evidence on the safety of PTX and make recommendations on the role PTX devices in the management of patients with PAD. The EAG determined that PTX devices could still be used in patients with CLTI, but only in the context of an RCT. The EAG specifically endorsed the reopening of BASIL-3 to recruitment (which occurred on 16 September 2019). The EAG recommendation was accepted by the MHRA and NIHR HTA on the condition that this supplementary analysis should be performed pooling the drug arms (DCBA ± BMS DES) in BASIL-3 to compare clinical outcomes, specifically mortality rate, with those participants who received only PBA ± BMS. This further analysis is the subject of this current report.

## Methods

### Study design and participants

The BASIL-3 methods have previously been described in full^5^. In short, BASIL-3 was a three-arm, multicentre, superiority, phase 3 RCT trial conducted in 35 UK National Health Service (NHS) vascular units. Eligible participants were those who presented with CLTI (ischaemic rest pain and/or tissue loss) in whom a multidisciplinary team (MDT) of vascular surgeons and interventional radiologists determined that an FP ± IP endovascular first revascularization strategy was preferred to surgical bypass. Participants were randomized 1:1:1 using a secure online system to receive either FP PBA (± BMS), DCBA (± BMS) or primary DES as their first revascularization strategy. For this prespecified supplementary analysis, we pooled data from the DCBA (± BMS) and DES arms to form a single ‘drug technologies’ (DT) group and compared those data against data from the PBA (± BMS) group. Participants were followed up locally at 1 month following their first revascularization procedure, and then at 6, 12, and 24 months and annually post-randomization until the last recruited participant had been followed for 24 months. In England and Wales, death and major amputation data were obtained until the end of follow-up from NHS Digital (the statutory custodian for health and social care data for England and Wales).

### Outcomes

The primary outcome for this supplementary analysis was overall survival (OS), defined as the time from randomization to death from any cause. The secondary outcomes included AFS, major amputations, major adverse limb events (MALE), major adverse cardiovascular events (MACE), further interventions, and 30-day mortality and morbidity rate. AFS was defined as the time to major (above-ankle) amputation of the trial leg or death from any cause, whichever occurred first. MALE comprised further revascularization of the trial leg or major amputation of the trial leg. MACE comprised a new diagnosis of CLTI in the contralateral leg, major amputation of the contralateral leg, myocardial infarction, stroke, and transient ischaemic attack. Further interventions captured any additional revascularization procedures to the trial leg. Thirty-day mortality and morbidity rates comprised deaths and significant complications (including serious adverse events) occurring within 30 days of the first trial intervention.

### Statistical analysis

The statistical methods employed in this supplementary analysis are consistent with the methods used in the main BASIL-3 trial analysis and were pre-specified in a statistical analysis plan, with the key difference being the pooling of DCBA (± BMS) and DES into a DT group. Unlike BASIL-3 where two separate comparisons (PBA ± BMS *versus* DCBA ± BMS and PBA ± BMS *versus* DES) were made, as we are only making one comparison (DT *versus* PBA ± BMS) here, we present one-sided 95% confidence intervals rather than the two-sided 97.5% confidence intervals presented in the main BASIL-3 analysis. Although this analysis was prespecified, it was not the primary objective of the BASIL-3 trial, and so, we do not conduct formal hypothesis testing or present values of *P*.

Baseline characteristics were summarized by group using means and standard deviations or medians and interquartile ranges for continuous variables, and numbers and frequencies for categorical variables. Time to event outcomes were analysed using Cox proportional hazard regression to estimate a hazard ratio (HR), and Kaplan–Meier survival curves were produced for visual representation of survival probabilities. For OS, Kaplan–Meier event rates were calculated at 1, 2, and 5 years to match the time points presented in the Katsanos meta-analysis. Further prespecified analyses were conducted using flexible parametric models with time-varying covariates for treatment, to consider the effects of non-proportional hazards in time to event outcomes. Where the outcome had competing risks, cause-specific Cox models and Fine–Gray models were fitted to estimate cause-specific HRs (CSHR) and subdistribution HRs (SHR) respectively, and cumulative incidence plots were produced. Binary outcomes were analysed with binomial generalized linear models with a log and identity link to estimate risk ratios (RR) and risk differences (RD) respectively. All regression models were adjusted for minimization variables as fixed effects, except the randomizing vascular unit (site) which was included as a random effect (or shared frailty variable for time to event outcomes). If fully adjusted models were not possible due to difficulty with convergence, unadjusted estimates were produced. All analyses were conducted using the intention-to-treat (ITT) principle, with all participants having been analysed according to the arms to which they had been randomized. A supplementary per-protocol analysis including only participants who received the randomized device to the FP segment for their first post-randomization revascularization was conducted for OS and AFS only.

## Results

Between 29 January 2016 and 26 August 2019, 481 participants were randomized to PBA ± BMS (*n* = 160), DCBA ± BMS (*n* = 161), or DES (*n* = 160). One participant was randomized to the DES arm without written informed consent. So, their data were removed, and they are not included in the analyses. Baseline characteristics of participants are displayed in *Table 1*. Most drug devices used in BASIL-3 were PTX based. One patient received a sirolimus coated DCB and eight received everolimus DES.

**Table 1:** Baseline characteristics by group

	Drug technologies *N* = 320* (%)	Plain balloon angioplasty *N* = 160 (%)
**Age^†^ (years)**		
≤60	50 (16%)	28 (18%)
61–70	91 (28%)	46 (29%)
71–80	109 (34%)	55 (34%)
>80	70 (22%)	31 (19%)
Mean(s.d.)	71.9(10.9)	71.5(10.6)
**Gender†**		
Male	208 (65%)	105 (66%)
Female	112 (35%)	55 (34%)
**Diabetes†**		
Yes	175 (55%)	89 (56%)
No	145 (45%)	71 (44%)
**Chronic kidney disease†,‡**		
Yes	109 (34%)	53 (33%)
No	211 (66%)	107 (67%)
**Severity of clinical disease†**		
Rest/night pain only	79 (25%)	41 (26%)
Tissue loss	58 (18%)	31 (19%)
Both	183 (57%)	88 (55%)
**Artery intended for treatment†**		
Superficial femoral only	205 (64%)	102 (64%)
Popliteal only	27 (8%)	14 (9%)
Both	88 (28%)	44 (28%)
**Previous permissible intervention to the trial leg†**		
Yes	57 (18%)	23 (14%)
No	263 (82%)	137 (86%)
**Hybrid procedure planned†**		
Yes	27 (8%)	16 (10%)
No	293 (92%)	144 (90%)
**Trial leg**		
Right	157 (49%)	79 (49%)
Left	163 (51%)	81 (51%)
**BMI (kg/m^2^)**		
Mean(s.d.)	27.3(6.5)	27.6(6.0)
Missing	30	13
**eGFR (ml/min/1.73 m^2^)**		
Mean(s.d.)	65.7(23.2)	65.9(23.7)
**Mobility**		
Fully ambulant without walking aid	125 (39%)	67 (42%)
Ambulant with walking aid	165 (52%)	80 (50%)
Wheelchair bound	27 (8%)	13 (8%)
Bed bound	3 (1%)	0 (−)
**Smoking status**		
Never	51 (16%)	34 (21%)
Ex	177 (55%)	91 (57%)
Current	91 (29%)	35 (22%)
Missing	1	0
**Pack years (in ex or current smokers)**		
Mean(s.d.)	38.4(30.6)	36.0(23.7)
Missing	44	21
**Ethnicity**		
White	300 (94%)	151 (94%)
Black/Black British	6 (2%)	5 (3%)
Asian/Asian British	11 (3%)	3 (2%)
Mixed	2 (1%)	0 (−)
Chinese or other ethnic group	0 (−)	1 (1%)
Missing	1	0
**Medical history**		
Stroke	45 (14%)	24 (15%)
Myocardial infarction (MI)	61 (19%)	34 (21%)
Missing	2	0
Angina	50 (16%)	28 (18%)
Missing	4	0
Coronary artery bypass graft (CABG)	35 (11%)	20 (13%)
Percutaneous coronary intervention (PCI)	33 (10%)	18 (11%)
Missing	3	1
Dialysis	15 (5%)	7 (4%)
Missing	1	0
**Imaging method**		
Duplex ultrasound only	112 (35%)	58 (36%)
MRA only	34 (11%)	21 (13%)
CTA only	93 (29%)	43 (27%)
DSA only	18 (6%)	8 (5%)
Duplex ultrasound + MRA	13 (4%)	12 (8%)
Duplex ultrasound + CTA	33 (10%)	10 (6%)
Duplex ultrasound + DSA	14 (4%)	6 (4%)
CTA + DSA	1 (<1%)	1 (1%)
No imaging	1 (<1%)	0 (−)
Missing	1	1
**Medical therapy**		
Antiplatelets and anticoagulants		
Both	44 (14%)	18 (11%)
Only antiplatelet	195 (61%)	100 (63%)
Only anticoagulant	42 (13%)	20 (13%)
Neither	39 (12%)	22 (14%)
Aspirin	148 (46%)	77 (49%)
Missing	0	2
Clopidogrel	112 (35%)	55 (35%)
Missing	0	3
Warfarin	29 (9%)	18 (11%)
Missing	3	2
Any other antiplatelet or anticoagulant	70 (23%)	30 (19%)
Missing	10	5
**Analgesics**		
Any analgesic	261 (83%)	119 (76%)
Missing	6	3
Paracetamol	212 (67%)	98 (62%)
Missing	2	1
Opiates	167 (53%)	60 (38%)
Missing	3	3
Non-steroidal anti-inflammatory drugs	26 (8%)	9 (6%)
Missing	9	1
Gabapentin/pregabalin	65 (21%)	30 (20%)
Missing	16	8
Amitriptyline	43 (14%)	19 (12%)
Missing	3	2
**Other medical therapy**		
Treatment for hypercholesterolaemia	240 (75%)	124 (79%)
Missing	0	4
Treatment for hypertension	237 (75%)	116 (76%)
Missing	3	7
**Previous vascular intervention to the trial leg§**		
Endovascular	44 (14%)	19 (12%)
Surgery	22 (7%)	6 (4%)
Minor amputation	24 (8%)	7 (4%)
Missing	0	1
**Previous vascular intervention to the non-trial leg§**		
Endovascular	50 (16%)	31 (19%)
Surgery	30 (9%)	13 (8%)
Minor amputation	19 (6%)	8 (5%)
Below knee amputation	10 (3%)	5 (3%)
Above knee amputation	10 (3%)	3 (2%)
Missing	1	0

CTA, computed tomography angiography; DSA, digital subtraction angiography; eGFR, estimated globular filtration rate; MRA, magnetic resonance angiograph. *Excluding the participant who did not provide consent. †Minimization variables used in the BASIL-3 randomisation algorithm. ‡CKD will be defined as stage 3 or worse based on estimated GFR of <60 (ml/min/1.73 m^2^). §Not mutually exclusive.

**Fig. 1:**
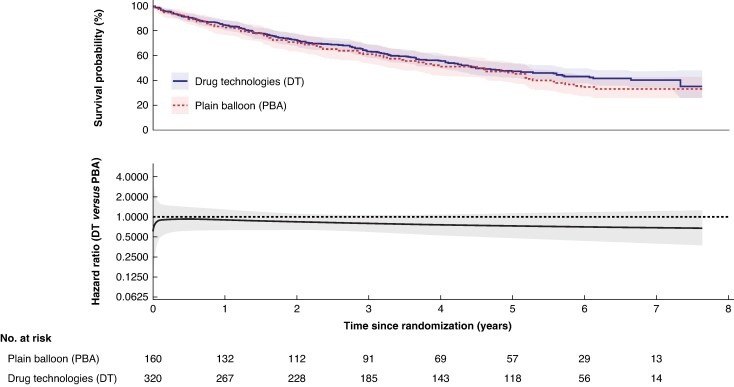
Overall survival—intention to treat analysis

**Fig. 2:**
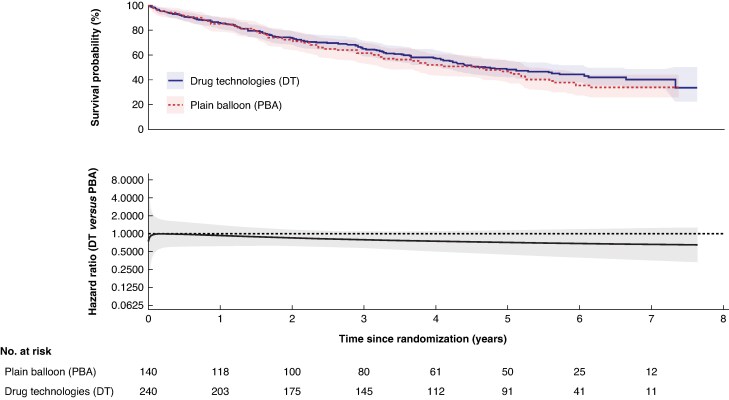
Overall survival—per protocol analysis

The median length of follow-up in surviving participants at the end of follow-up was 5.6 years. The median survival in the DT group was 4.5 years (95% c.i.: 4.0 years to 5.7 years), compared with 4.6 years (95% c.i.: 3.2 years to 5.2 years) in the PBA group. In the DT group, 170/320 (53%) participants died from any cause during follow-up, compared with 96/160 (60%) in the PBA ± BMS group (adjusted HR: 0.83; 95% c.i.: 0.64 to 1.07, *Fig. 1*); this was consistent in the per-protocol analysis (*Fig. 2*). The model was checked to assess the non-proportional hazard assumption, and this was found to be violated. A flexible parametric model was fitted and a plot of the HR over time with 95% c.i. is provided in *Fig. 1* and *2*.

**Fig. 3:**
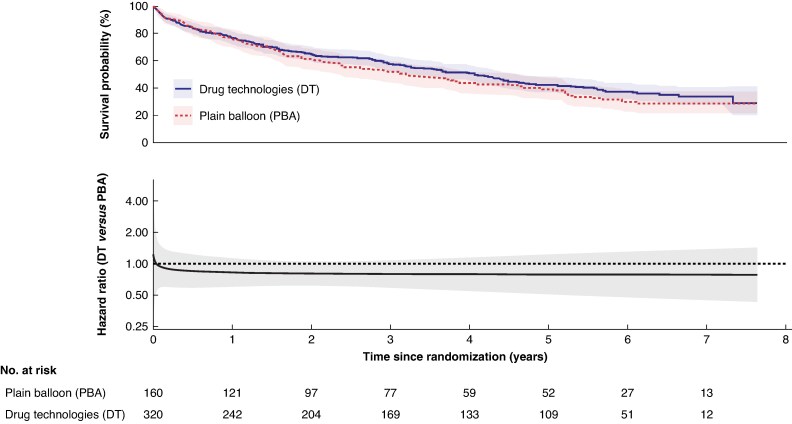
Amputation-free survival—intention to treat analysis

**Fig. 4:**
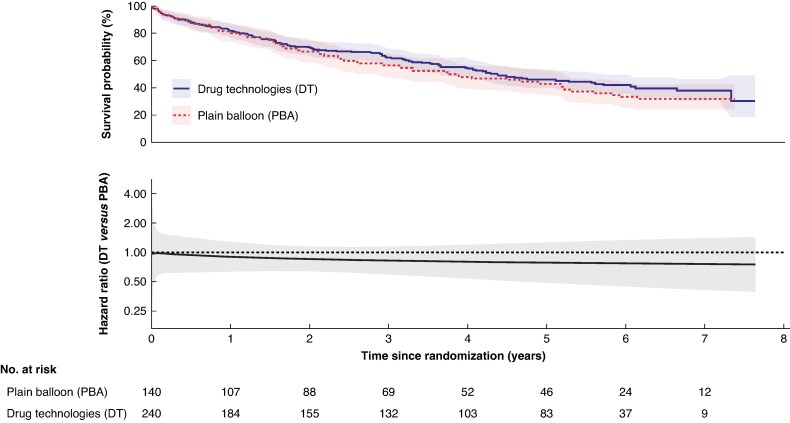
Amputation-free survival—per protocol analysis

In the DT group, 190/320 (59%) participants had a major amputation or died (no AFS), compared with 106/160 (66%) in the PBA ± BMS group (adjusted HR: 0.84; 95% c.i.: 0.66 to 1.06, *Fig. 3*); this was consistent in the per-protocol analysis (*Fig. 4*). The model was checked to assess the non-proportional hazard assumption, and this was found to be violated. A flexible parametric model was fitted and a plot of the HR over time with 95% c.i. is provided in *Fig. 4*.

In the DT arm, 43/320 (13%) participants had a major amputation compared with 23/160 (14%) in the PBA ± BMS group (adjusted CSHR: 0.90, 95% c.i.: 0.54 to 1.50; SHR 0.93: 95% c.i.: 0.56 to 1.53). There were no differences reported for the other secondary outcomes (*Table 2*).

**Table 2:** Primary and secondary outcomes by group

	Drug technologies *N* = 320* (%)	Plain balloon angioplasty *N* = 160 (%)	Estimate (95% c.i.)
**Primary outcome**			
Death from any cause (ITT)	170 (53%)	96 (60%)	HR: 0.83 (0.64 to 1.07)‡
1 year†	50, 267 (16%)	27, 132 (17%)	
2 years†	88, 228 (28%)	47, 112 (30%)	
5 years†	158, 117 (53%)	83, 57 (54%)	
Death from any cause (per protocol)	127/140 (53%)	83/140 (59%)	HR: 0.83 (0.62 to 1.10)‡
**Secondary outcomes**			
No AFS (ITT)	190 (59%)	106 (66%)	HR: 0.84 (0.66 to 1.06)‡
No AFS (per protocol)	145/240 (60%)	91/140 (65%)	HR: 0.87 (0.66 to 1.14)‡
Major amputation	43 (13%)	23 (14%)	CSHR: 0.90 (0.54 to 1.50)‡ SHR: 0.93 (0.56 to 1.53)^§^
Further interventions	90 (28%)	48 (30%)	RR: 0.95 (0.72 to 1.26)‡ RD: −0.016 (−0.101 to 0.070)¶
MALE	113 (35%)	59 (37%)	RR: 0.95 (0.72 to 1.26)‡ RD: −0.020 (−0.109 to 0.068)¶ CSHR: 0.94 (0.69 to 1.29)‡ SHR: 0.98 (0.71 to 1.34)^§^
MACE	124 (39%)	49 (31%)	RR: 1.28 (0.98 to 1.68)‡ RD: 0.083 (−0.005 to 0.171)^#^ CSHR: 1.30 (0.93 to 1.81)‡ SHR: 1.40 (1.00 to 1.94)^§^
30-day morbidity	104 (33%)	45 (28%)	RR: 1.17 (0.88 to 1.55)^§^ RD: 0.044 (−0.043 to 0.130)**
30-day mortality	6 (2%)	7 (4%)	RR: 0.41 (0.13 to 1.25)^§^ RD: −0.025 (−0.060 to 0.010)**

AFS, amputation-free survival; CSHR, cause-specific hazard ratio; HR, hazard ratio; ITT, intention to treat; MACE, major adverse cardiovascular events; MALE, major adverse limb events; RD, risk difference; RR, risk ratio; SHR, subdistribution hazard ratio. *Excluding the participant who did not provide consent. †Number of deaths, number at risk (Kaplan–Meier probability of death). ‡Adjusted for age, gender, diabetes mellitus, chronic kidney disease, severity of clinical disease, previous intervention to the trial leg, intention for a hybrid procedure, intended artery for treatment and centre. Values <1 favour drug technologies. §Adjusted for age, gender, diabetes mellitus, chronic kidney disease, severity of clinical disease, previous intervention to the trial leg, intention for a hybrid procedure and intended artery for treatment. Values <1 favour drug technologies. ¶Adjusted for age, gender, diabetes mellitus, chronic kidney disease, severity of clinical disease, previous intervention to the trial leg, intention for a hybrid procedure, intended artery for treatment and centre. Values <0 favour drug technologies. #Adjusted for age, gender, diabetes mellitus, chronic kidney disease, severity of clinical disease, previous intervention to the trial leg, intention for a hybrid procedure and intended artery for treatment. Values <0 favour drug technologies. **Unadjusted. Values <0 favour drug technologies.

## Discussion

The main finding of the current analysis is that in the BASIL-3 RCT there was no evidence of increased mortality with the use of drug-based (PTX) technologies when compared to PBA ± BMS. In the ITT analysis, the most likely effect was a 17% reduced rate of death in those randomized to DT, but the wide confidence interval supported effects between a large reduction (HR: 0.64) and a small increase (HR: 1.07) in death from any cause. This was consistent with the per-protocol analysis. At 5 years, the time point where the largest difference in mortality was seen in the Katsanos meta-analysis, all-cause mortality in the DT and PBA ± BMS groups was almost identical (53% *versus* 54% respectively). These findings were consistent with the results of the primary BASIL-3 analysis, where there were no differences in mortality between any of the three randomized groups individually^6^. When considering such a comparison it is important to highlight several important points: first, the BASIL-3 trial only included patients with CLTI and not those with IC; second, patients were selected for endovascular treatment based on decisions made with an MDT and not according to lesion morphology or length; and, third, that BASIL-3 allowed the use of any UK-licensed drug-eluting technology and did not specify device or dosage.

The Katsanos meta-analysis, although imperfect due to the quality of studies collated, fuelled an intense international debate regarding the safety of PTX in patients with PAD. Since then, several groups have published work that suggests there is no increased mortality rate with the use of PTX in similar cohorts of patients. A retrospective review of over 1000 patients with IC was published in 2020 and demonstrated a reduction in mortality rate with PTX-based treatment of FP lesions^8^ . More recently, a systematic review specifically focused on the risk of mortality with PTX in 19 RCTs performed since 2018 and found no increased risk of death in those participants who received PTX out to 5 years^9^. A further individual patient data meta-analysis of 10 RCTs also found no difference in mortality rate between PTX and PBA (including both ITT and sensitivity analyses)^10^. These studies did not include data from BASIL-3. The US Vascular Quality Initiative has examined the outcomes of over 28 000 patients with CLTI (non-matched) who received PTX-based devices compared with PBA^4^. Those who received PTX had higher rates of limb salvage, primary patency, and freedom from MALE. At 54 months there was no difference in overall mortality rate between the groups.

The PTX debate in patients with IC has, however, recently reopened following the publication of the Swedish Drug Elution Trial in Peripheral Arterial Disease 2 (SWEDEPAD-2)^11^. In 22 centres in Sweden, 1155 participants were randomly assigned to PTX-coated devices or uncoated devices. Five-year mortality rate was higher in those allocated with PTX-coated devices by almost 50% (HR 1.47 (95% c.i. 1.09 to 1.98), *P* = 0.01). Interestingly, in the parallel SWEDEPAD-1, which randomized participants with CLTI to similar groups, no differences in mortality rate were reported (HR 1.04 (95% c.i. 0.92 to 1.17), *P* = 0.54)^12^.

As expected, given that all the patients in BASIL-3 had CLTI, the overall all-cause mortality rate was much higher than in the Katsanos meta-analysis and SWEDEPAD-2 and, therefore, the median follow-up and survival was short in comparison. When compared to those with IC, CLTI patients experience much higher competing risks from cardiovascular mortality and other smoking-related diseases. As such, many CLTI patients may not survive long enough to be affected by any difference in mortality that might be due to their exposure to PTX. Recent work has demonstrated that the great majority of ‘real-world’ CLTI patients, akin to those randomized in BASIL-3, would not be eligible for randomization into most previous industry-sponsored PTX trials, as most of these trials included IC patients, and also imposed strict inclusion criteria regarding lesion morphology^13^ .

As noted above, the great majority of the participants allocated to DT in BASIL-3 received PTX devices. Only nine did not; one received a sirolimus DCB and eight received everolimus DES. These patients accounted for 1.9% of the total cohort and 2.8% of those randomized to DT. The impact of these newer DT, in this patient cohort, is currently unknown.

### Strengths and limitations

This further pooled analysis was prespecified by the funder and the UK regulatory body MHRA following the suggestion that DT-based devices increased the risk of death when used in patients with PAD. These data from a UK pragmatic RCT from multiple specialist centres have demonstrated that in this specific patient population there does not appear to be an increased risk of death from such technology. This is the first UK-based trial to analyse such outcomes from RCT data in a pooled fashion. The BASIL-3 trial was powered to assess AFS in three individual groups of CLTI patients (PBA ± BMS, DCB ± BMS, and DES alone). Here we have examined pooled overall survival in the DT groups (DCB ± PBA and DES *versus* PBA ± BMS). BASIL-3 was not powered to assess this outcome in this group and these results must be interpreted with that in mind.

The strengths and limitations of the primary analysis of the BASIL-3 trial have been discussed at length elsewhere^6^. BASIL-3 was robust and representative of the majority of UK practice at the time trial enrolment was active. Newer adjunctive technologies such as intravascular ultrasound, non-PTX DT, and biomimetic stents were barely used in BASIL-3 or unavailable. The impact of these new technologies on outcomes are still unknown, but any impact they may have seems more likely to influence technical outcomes and possibly limb salvage rather than all-cause mortality rate. One criticism of the BASIL-3 trial was that there was a higher frequency of patients who did not receive their allocated intervention in the DT group. However, here we report consistent findings between the intention to treat analysis and those treated per protocol.

It may be considered a limitation (or alternatively a strength) that in BASIL-3 all DT (which were overwhelmingly PTX based) devices were assumed to have a class effect. It is possible that different drugs, excipients, or devices may have differing properties that produce different results when viewed in isolation. However, there is no evidence to support those notions. BASIL-3 was not powered to demonstrate this, and as such, an analysis of individual devices was not done.

The impact of BASIL-3 and the data presented here on the use of DT is uncertain, whereas a link between mortality and device use has not been demonstrated, like SWEDEPAD-1 we have not demonstrated clinical benefits over PBA. Further clarification is required regarding the clinical and cost effectiveness of adjunctive devices designed to optimize the efficacy of endovascular intervention in the femoropopliteal arteries.

## Collaborators

A. Ganeshan (Heartlands Hospital, University Hospitals Birmingham, Birmingham, UK); H. Zayed (Guys & St Thomas' Hospital, London, UK); R. Davies (Glenfield Hospital, University Hospitals Leicester, Leicester, UK); A. Saratzis (Glenfield Hospital, University Hospitals Leicester, Leicester, UK); I. Chetter (Hull University Teaching Hospitals NHS Trust, Hull, UK); S. Butterfield (Wythenshawe Hospital, Manchester University NHS FT, Manchester, UK); S. Goode (Northern General Hospital, Sheffield Teaching Hospitals NHS FT, Sheffield, UK); J. Metcalfe (Dorset County Hospital NHS FT, Dorset, UK); P. Mezes (Southmead Hospital, North Bristol NHS Trust, Bristol, UK); S. Hobbs (Russell's Hall Hospital, Dudley Group NHS FT, Dudley, UK); N. Goyal (Aneurin Bevin University Health Board, Royal Gwent Hospital, Newport, Wales); J. Patel (Leeds Teaching Hospitals NHS Trust, Leeds, UK); M. Caruna (Royal Sussex Hospital, University Hospitals Sussex NHS FT, Brighton, UK); T. Rashid (Manchester Royal Infirmary, Manchester University NHS FT, Manchester, UK); N. Das (Kent & Canterbury Hospital, East Kent Hospitals NHS FT, Canterbury, UK); N. Arya (Pilgrim Hospital Boston, United Lincolnshire Hospitals NHS Trust, Boston, UK); P. Chong (Frimley Park Hospital, Frimley Health NHS FT, Camberley, UK); S. Habib (Queen's Medical Centre, Nottingham University Hospitals NHS Trust, Nottingham, UK); R. White (University Hospital Wales, Cardiff & Vale University Health Board, Cardiff, Wales); G. Antoniou (Royal Oldham Hospital, Northern Care Alliance NHS FT, Oldham, UK); A. Davies ((St Marys Paddington, Imperial College Healthcare NHS Trust, London, UK); A. Rogers (Royal Cornwall Hospital NHS Trust, Truro, UK); S. Dindyal (Basildon University Hospital, Mid and South Essex NHS FT, Basildon, UK); N. Arestis (Forth Valley Royal Hospital, NHS Forth Valley, Larbert, Scotland); G. Weir (Royal Infirmary Edinburgh, NHS Lothian, Edinburgh, Scotland); P. Davey (County Durham & Darlington NHS FT, Darlington, UK); R. Das (St George's University Hospitals NHS FT, London, UK); G. Stansby (Freeman Hospital, Newcastle upon Tyne Hospitals NHS FT, Newcastle upon Tyne, UK); R. Eifell (Cumberland Infirmary, North Cumbria Integrated NHS FT, Carlisle, UK); J. Asquith (Royal Stoke University Hospital, University Hospitals of North Midlands NHS Trust, Stoke on Trent, UK); L. Wijesinghe, Royal Bournemouth Hospital, University Hospital Dorset, Bournemouth, UK); A. Chaudhuri (Bedford Hospital NHS Trust, Bedford, UK); J. Sathianathan (Dumfries & Galloway Royal Infirmary, NHS Dumfries & Galloway, Dumfries, Scotland); N. Thayur (Colchester Hospital, East Suffolk & North Essex NHS FT, Colchester, UK); F. Ahmad (Royal Berkshire Hospital, Royal Berkshire NHS FT, Reading, UK).

## Data Availability

Requests for data should be directed to the corresponding author. Requests will be assessed for scientific rigour before being granted. Data will be anonymized and securely transferred. A data sharing agreement might be required.
